# Psychocultural experiences of medical students in simulated care in cases of type 2 diabetes mellitus at a public university in southeastern brazil: A qualitative study

**DOI:** 10.1192/j.eurpsy.2021.1720

**Published:** 2021-08-13

**Authors:** E. Turato, G. Lavorato-Neto, M.C. Parisi

**Affiliations:** 1 Medical Psychology And Psychiatry, University of Campinas, Campinas, Brazil; 2 Lpcq - Laboratory Of Clinical-qualitative Research, University of Campinas, Campinas, Brazil

**Keywords:** Medical Education, type 2 diabetes mellitus, medical psychology, Qualitative Research

## Abstract

**Introduction:**

The generalist assistance at the Primary Attention is fundamental to face the increase of type 2 diabetes mellitus cases through the relationship physician-patient. This sets the therapeutic plan and its continuous review. Therapeutic Plan could be affected by the same psychocultural phenomena related to the increasing cases numbers of DM2. Therefore, new trends in Medical Psychology have been promoted during medical undergraduate course. These incorporate methods and concepts of Liberal Arts to develop specific psychosocial management skills to DM2 clinic.

**Objectives:**

AIM: To understand the experience of medical students in the simulated care of DM2 cases in two different moments: 1) to diagnose and start treatment; 2) start insulinization.

**Methods:**

METHOD: Clinical-Qualitative design; data collected through an semidirected interview of open-end questions in depth; thematic analysis generated categories discussed in light of Medical Psychology of psychodynamic framework.

**Results:**

RESULTS: Ten clinical clerkship students attended as clinicians two cases of Standard Patients of DM2. They reported their reflections toward the role they should sustain: being doctor in front of the patient and their colleagues; difficulty to play the leading and show skills and knowledge in a scenario full of surprises and fantasies; an existential and professional gains in simulation activity; and considerations about responsible in conduct so impacting situations to patients.

**Conclusions:**

FINAL CONSIDERATONS: The themes translate moments during their simulated attending experience in which they have not sustained their semblance – the intended rule. These could be enriched though group reflecting, supervisor discussion, and patient dialog in the process of developing Medical Psychology skills.

**Disclosure:**

No significant relationships.

